# Salience of Somatosensory Stimulus Modulating External-to-Internal Orienting Attention

**DOI:** 10.3389/fnhum.2017.00428

**Published:** 2017-08-24

**Authors:** Jiaxin Peng, Sam C. C. Chan, Bolton K. H. Chau, Qiuhua Yu, Chetwyn C. H. Chan

**Affiliations:** Applied Cognitive Neuroscience Laboratory, Department of Rehabilitation Sciences, The Hong Kong Polytechnic University Kowloon, Hong Kong

**Keywords:** attention, orienting, somatosensory stimulus, salience of stimulus, external attention, internal attention

## Abstract

Shifting between one’s external and internal environments involves orienting attention. Studies on differentiating subprocesses associated with external-to-internal orienting attention are limited. This study aimed to reveal the characteristics of the disengagement, shifting and reengagement subprocesses by using somatosensory external stimuli and internally generated images. Study participants were to perceive nociceptive external stimuli (External Low (E_L_) or External High (E_H_)) induced by electrical stimulations (50 ms) followed by mentally rehearsing learned subnociceptive images (Internal Low (I_L_) and Internal High (I_H_)). Behavioral responses and EEG signals of the participants were recorded. The three significant components elicited were: fronto-central negativity (FCN; 128–180 ms), fronto-central P2 (200–260 ms), and central P3 (320–380 ms), which reflected the three subprocesses, respectively. Differences in the FCN and P2 amplitudes during the orienting to the subnociceptive images revealed only in the E_H_ but not E_L_ stimulus condition that are new findings. The results indicated that modulations of the disengagement and shifting processes only happened if the external nociceptive stimuli were of high salience and the external-to-internal incongruence was large. The reengaging process reflected from the amplitude of P3 correlated significantly with attenuation of the pain intensity felt from the external nociceptive stimuli. These findings suggested that the subprocesses underlying external-to-internal orienting attention serve different roles. Disengagement subprocess tends to be stimulus dependent, which is bottom-up in nature. Shifting and reengagement tend to be top-down subprocesses, which taps on cognitive control. This subprocess may account for the attenuation effects on perceived pain intensity after orienting attention.

## Introduction

Orienting attention is crucial for enabling individuals to perceive information from the external environment for internal processing such as discrimination and decision making (Lepsien and Nobre, 2006; Chun et al., 2011). The external-to-internal process involves disengagement from the external stimuli followed by engagement with the internal representations generated in the mind. Posner’s model of orienting attention (Posner et al., 1984; Petersen and Posner, 2012) theorized three steps of orienting attention between two stimuli; namely, disengagement, shifting and reengagement. The three-step model, however, elucidates orienting only between two external stimuli but not from external stimuli to internal representations. The external-to-internal orienting process is important as information processing in humans mostly requires encoding information available in the external environment for other internal processes, but its mechanism is still not clear.

Previous studies focused on orienting attention on external stimulus (called External Attention, EA) or internal stimulus (called Internal Attention, IA) predominantly involving visual modality. Griffin and Nobre (2003) reported that behavioral performances were improved (e.g., faster reaction time and higher accuracy rate) when participants’ orienting attention to the cued spatial location was enhanced in the EA as well as the IA condition. Nevertheless, previous studies indicated that EA and IA involved different neural processes. Sauce et al. (2014) articulated their functionality in that EA primarily inhibits the perception of distractions and noises associated with external stimulus, whereas IA facilitates the perception against interferences associated with internal representation such as irrelevant memory episodes. Griffin and Nobre (2003) demonstrated that EA and IA differed in the N1 component, of which IA elicited more negative-going waveforms at the frontal (120–200 ms) and central regions (160–200 ms) than EA. Tanoue et al. (2012) reported that performance involving IA was found to be more affected than that of EA when the frontal lobe was under stimulation. Taken together, orienting attention to an internal stimulus involves more frontal control than orienting attention to an external stimulus.

Studies on orienting attention involving somatosensory modality further disentangle the subprocesses associated with external stimuli, but also external-to-internal stimuli. Fronto-centrally distributed N1 (also called FCN; 100–200 ms) has been revealed as a common marker associated with orienting attention among external somatosensory stimuli (Kida et al., 2004; Dowman, 2007, 2011; Dowman et al., 2007). In particularly, less negative-going frontal N1 was associated with the disengaging process among external somatosensory stimuli presented at different spatial locations (Katus et al., 2012), as well as from a somatosensory to a visual stimulus (Ohara et al., 2006; Staines et al., 2014) or vice versa (Dowman, 2007, 2011; Dowman et al., 2007). Besides the N1, Dowman (2007, 2011) proposed that more P2 (260–380 ms) elicited at the fronto-central region was related to shifting attention, whereas P3a (320–390 ms) elicited at the centro-parietal region was related to reengagement. Taken together, FCN, fronto-central P2 and central P3a appear to play different roles when an individual disengages, shifts and reengages one’s attention on external somatosensory stimulus.

All the studies reviewed above focused on EA processes. Only a handful of studies addressed neural processes of IA and external-to-internal orienting attention involving somatosensory modality. For instance, Legrain et al.’s (2013) reported that working memory modulated the external-to-internal orienting process. The external stimuli used were nociceptive (by CO_2_ laser) or subnociceptive stimulations (by electrical stimulations), and the internal presentations were images of visual dots (Legrain et al.’s 2013). The external-to-internal shifting processes were associated with less negative-going N1 and less positive-going P2 elicited in the frontal and central regions, respectively. Legrain et al.’s (2013) proposed the N1 component reflected attending to and disengaging from the external nociceptive stimulus, whereas the P2 component reflected the shifting process. The involvement of the centrally distributed P2 in the external-to-internal shifting process was further corroborated by the results revealed in Chan et al.’s (2012) study, which used nociceptive stimulations as the external stimuli and subnociceptive images as the internal representations. Their results, however, are different from those reported by Dowman (2007, 2011), which suggested shifting was associated with FCN, P2 and P3a. The discrepancies in the results further suggest that orienting attention to somatosensory stimuli involves unique neural processes depending on the external or internal environment in which the stimuli are processed.

This study aimed to differentiate the subprocesses associated with external-to-internal orienting attention. In particular, we attempted to reveal the characteristics of the disengagement, shifting and reengagement subprocesses by using somatosensory external stimuli and internally generated images. The external stimuli involved were nociceptive stimulations to be perceived by the participants and the internal representations were subnociceptive images generated by the participants after receiving training. The external-to-internal perceptual processes required the participants to perceive different levels of salience of nociceptive stimulations followed by generating a predetermined subnociceptive image of specific salience level. We hypothesized that perception of more salient nociceptive external stimuli would result in a higher level of bottom-up control for initiating a disengagement process, which would yield an FCN (an earlier component). The shifting to more salient internal subnociceptive images would result in a higher level of top-down control for the shifting and reengagement processes, which would yield fronto-central P2 and centro-parietal P3 (later components). The external-to-internal orienting attention would associate with attenuation of the pain intensity felt by the participants for the external nociceptive stimulations.

## Materials and Methods

### Participants

Twenty-two healthy participants (13 females) were recruited. Their mean age was 39.0 (SD = 11.6). All of them had a high school education or above and scored within the norms on the Stroop Test (measure of executive control functions). The participants did not report any type of pain in the past 6 months. The purpose of the study was explained to and informed consents were obtained from each participant. This study was approved by the research committee of the Department of Rehabilitation Sciences of The Hong Kong Polytechnic University.

### Pre-Experimental Preparation

#### Electrical Stimuli

The nociceptive and subnociceptive stimuli used in the pre-experiment training and the experiment were 50-ms electrical stimulations at different intensity levels (25-pulse train of electrical square-wave pulses with 0.5-ms pulse duration and 500 Hz frequency) generated from an S88K Dual Output Square Pulse Stimulator (Grass Technologies, Grass-telefactor, West Warwick, RI, USA) and controlled by a constant current unit (CCU). These devices were the same as those used in Chan et al.’s (2012) study. Anode and cathode electrodes of the stimulator were attached to the skin of a flat bodily area posterior to the lateral malleolus of the left ankle and along the distribution of the sural nerve (L5-S1 dermatome; Dowman, 2007). The external nociceptive and subnociceptive stimuli were applied to this site during the pre-experiment training, and only the nociceptive stimuli were applied during the actual experiment.

#### Calibration of Stimuli

The procedure used for calibrating the nociceptive (i.e., painful) and subnociceptive (i.e., not painful) stimuli followed the sequential stepping-up and stepping-down method adopted in previous studies (De Pascalis et al., 2008; Chan et al.’s 2012). Three critical sensory thresholds were calibrated for the electrical stimulations generated for each participant’s minimum detectable sensation (MDS; the weakest stimulation intensity level with which participants detected a tactile sensation), just painful sensation (JPS; the minimal intensity with which participant’s perceived stimulation as painful and rated “1” on the 11-point numeric rating scale, or NRS; Jensen et al., 1986; Williamson and Hoggart, 2005) and very painful sensation (VPS; the intensity with which participants perceived a stimulation as very painful and rated “7” on the NRS). The mean voltages of the MDS, JPS (NRS = 1), and VPS (NRS = 7) of the participants were 3.32 mA (SD = 5.24 mA), 19.11 mA (SD = 6.38 mA), and 41.27 mA (SD = 12.60 mA), respectively (Table 1). For each participant, two levels (one-third and two-thirds) of intensity of the subnociceptive stimuli were determined between the MDS and the JPS (labeled as SN_L_ and SN_H_) and six levels of intensity of the nociceptive stimuli were determined by means of even distribution between the JPS and the VPS (labeled as L1 to L6).

**Table 1:** Mean (SD) of intensity, voltages and NRS ratings for the nociceptive (six) and sub-nociceptive (two) stimuli.

		Sub-nociceptive		Nociceptive					
		SN_L_	SN_H_	L1	L2	L3	L4	L5	L6
Voltage	Mean	3.4	16.1	20.7	24.2	28.0	32.6	36.7	41.0
	SD	5.3	6.1	7.1	7.6	8.6	9.6	10.9	12.3
NRS	Mean	-	-	1.6	2.7	3.6	4.7	5.3	5.7
	SD	-	-	0.7	0.8	1.0	0.7	0.6	0.8

*Note: SN_L_ and SN_H_ refer to lower and higher intensity sub-nociceptive stimulus, respectively; L1 to L6 are nociceptive stimulus with L1 being the lowest intensity and L6 being the highest intensity. NRS (Numeric Rating Scale), ranged from 0 to 10, reflects the pain intensity felt by the participant from the stimuli*.

#### Training

The participants learned to assign NRS ratings to represent the pain intensity felt for the individualized nociceptive and subnociceptive stimuli. This was an important component in the study as the participants were required to give an NRS rating of the pain intensity level of the brief external nociceptive stimulus (only 50 ms exposure) by the end of each trial. The training was to improve the validity and accuracy of the ratings to be assigned by the participants. Individualized calibrated nociceptive stimuli were randomly delivered to the lateral malleolar of the left leg after which the participants assigned one of the L1 to L6 ratings. There were at least 24 attempts and the training would end after the participants achieved at least 80% accuracy. In addition, as each trial involved generation of specific internal subnociceptive image (high or low) after perceiving the external nociceptive stimulus, the participants learned to associate the somatosensory sensation of the two subnociceptive stimuli with the high and low intensity descriptor. The same training protocol was used for learning the intensity level and association with the intensity descriptor of the nociceptive stimuli.

### Experimental Task

The design of the experimental task made reference to that employed in Chan et al.’s (2012). Among the six nociceptive stimuli, the three lower intensity stimuli were grouped into the E_L_ condition (low salience external stimuli) and the three higher intensity stimuli were grouped into the E_H_ condition (high salience external stimuli). Between-condition differences in the voltages of (*t*_(18)_ = 12.13, *p* < 0.001) and the NRS scores associated with the stimulation intensity (*t*_(18)_ = 16.14, *p* < 0.001) were statistically significant. The two internally generated subnociceptive image representations were categorized into I_L_ (internal low salience) and I_H_ (internal high salience), respectively.

**Figure 1:**
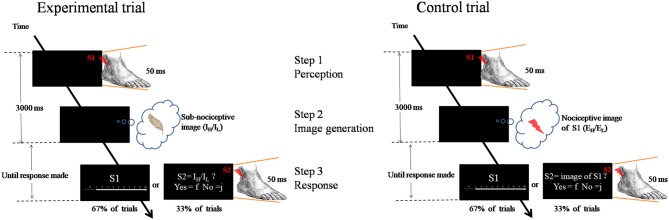
Schematic representation of the experimental paradigm. Left panel is an experimental trial in which the participant was to attend to an external nociceptive stimulus (S1, E_H_ or E_L_) and maintain the image (Step 1), and followed by generating a learnt sub-nociceptive image (I_H_ or I_L_; Step 2). During the response (Step 3), the participant was to assign a rating against Numeric Rating Scale (0 score = “non-painful” to 10 score = “extremely painful”) which represents the intensity of the pain felt for the S1 (in 67% of trials). In some occasions (33% of trials), the participant was to attend to a sub-nociceptive stimulus (S2) and judge whether the intensity of S2 was comparable with that of the sub-nociceptive image last generated. Right panel is a control trial which only contains Steps 1 and 3. In Step 3, the same procedure was followed except that, in some occasions, the S2 delivered to the participant was a nociceptive stimulus with 50% of the time the intensity was comparable to that of S1.

Each trial involved the participants’ engagement in three steps: (1) perception—perceiving an external nociceptive stimulus of 50 ms (S1); (2) image generation—generating a learned subnociceptive image; and (3) rating response—recalling the perceived external nociceptive stimulus (S1) and assigning an NRS rating reflecting the pain intensity felt from the nociceptive image (Figure 1).

A fixation cross (500 ms) was first presented at the middle of a computer monitor. After a varied interval of 1100–1300 ms, a 50 ms nociceptive stimulus (called S1)—randomized intensity (E_L_: L1–L3; E_H_: L4–L6)—would be delivered from the electrical stimulator at the lateral malleolus of the left ankle. A participant was to attend to the nociceptive stimulus (Step 1). Then, participants generated and rehearsed a learned subnociceptive image representation, SN_L_ or SN_H_, depending on the condition (Step 2). This process would require participants to disengage from the relatively strong external nociceptive stimulus, shift their attention and reengage with the relatively weak internally generated somatosensory image. The duration for steps 1 and 2 was 3000 ms. Participants were then asked to recall the pain felt from the external nociceptive stimulus and assign a numeric rating of the intensity of the sensation (Step 3). The response statement “How painful do you feel for S1?” appeared on the screen, which lasted until a response was received or after 6000 ms. The response was to press one of the 1-to-10 keys on a number keyboard that represented the perceived pain intensity. The control task required participants to engage in only steps 1 and 3, and skip Step 2. The participants were to perceive the external nociceptive stimulus, which lasted for 50 ms, and retain the image of the stimulus until end of 3000 ms. The same rating of the pain felt from the stimulus was conducted. As a validity check, one-third of the trials in each block involved participants judging the congruence between the internal image rehearsed with an external stimulus (S2) rather than rating the internal image as in Step 3 in the experiment. The S2 was a subnociceptive stimulation of intensity similar to I_H_ or I_L_ or a nociceptive stimulation of intensity equivalent to S1. The participants were to make responses to “Is the intensity of the stimulus similar to what you had in mind?”

The task was organized in a 2 × 2 (external stimuli × internal image representations) design. One High (E_H_) and one Low (E_L_) condition each manipulated the intensity of the external nociceptive stimuli to be perceived by the participants. Only the L4 to L6 stimuli were delivered in the E_H_ condition, and only L1 to L3 stimuli were delivered in the H_L_ condition. Additionally, one High (I_H_) and one Low (I_L_) condition each manipulated the intensity of the internally generated subnociceptive image representations. In the I_H_ condition, only the learned higher N_H_ image would be generated and rehearsed by the participants. The lower N_L_ image would be generated and rehearsed in the I_L_ condition. The 2 × 2 external-to-internal combinations produced a total of 324 trials. The trials were organized into three conditions (namely, I_H_, I_L_, and control) and three blocks were organized in each condition. Completion of 36 trials in each block took around 5 min. The sequence of stimuli presentation was counterbalanced across the participants, and the sequence of the blocks was organized in a pseudorandomized sequence.

### EEG Recording and Preprocessing

The EEG signals were recorded throughout tasks from scalps of the participants with a 64-channel cap based on the 10-20 system. The signals collected were preprocessed by CURRY Neuroimaging Suite software (Neuroscan, Compumedics Ltd., Abbotsford, VIC, Australia). Electrooculograph (EOG) was recorded by two pairs of electrodes located vertically and horizontally around the eyes to detect eye blinks and movements. The two reference electrodes were placed on the left and right mastoid. The EEG signal was sampled at 1024 Hz. All EEG/EOG electrode impedances were set to be less than 10 kΩ. The timing and presentation of all the stimuli were controlled by E-Prime (Psychology Software Tools, Inc.). During the preprocessing, all data were referenced to the average of the two referenced electrodes. The EEG was epoched from 200 ms before and 900 ms after the onset of the delivery of each external nociceptive stimulus. Ocular artifact reduction and a zero-pass filter with a low-pass of 30 Hz and 24 db/oct were applied.

### Data Analysis

Behavioral results were the NRS scores for the perceived pain intensity of the external nociceptive stimuli assigned by the participants by the end of each trial. Two-way, repeated-measures ANOVA was used to test the possible External (E_L_ vs. E_H_) and Internal (I_L_ vs. I_H_) effects on the NRS scores. *Post hoc* comparisons were conducted in the case of significant interaction effects on the External and Internal factors.

**Figure 2:**
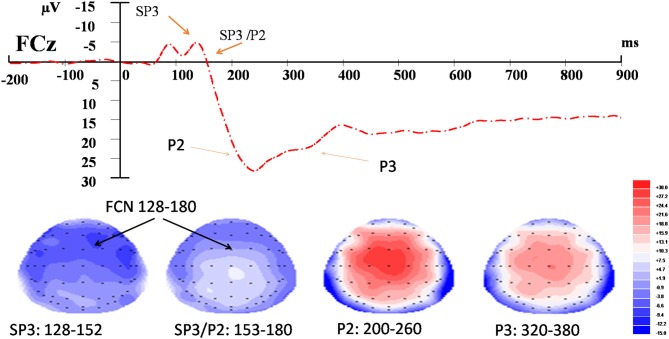
Event-related potential (ERP) waveform and topographic maps of the identified components. Upper panel: *t* = 0 corresponds to the onset of the nociceptive stimulus recorded at FCz. Bottom panel: topographic maps (top view) of amplitudes of fronto-central negativity (FCN; SP3, SP3/P2), P2 and P3 waves in their respective time-windows.

For the EEG data, the time windows of the components were determined according to the somatosensory-evoked potential method described in Dowman (2011). The onset and offset latencies of the negative potentials were first approximated by visual inspection. The stable period of a potential was the time window between the onset and offset latencies and was verified by employing the *r*^2^ statistics derived from the amplitudes of the potential captured from the 29 scalp electrodes. A stable period included the time points at which the *r*^2^ of the amplitudes between the midpoint (or peak) and that of the time point was ≥0.85. According to this method, the time windows for each of the three components were: 128–152 ms and 152–180 ms for the SP3 and SP3/P2 (respectively), 200–260 ms for the P2, and 320–380 ms for the P3 (Figure 2). Three-way, repeated-measures ANOVA was conducted to test the effects of External (E_L_ vs. E_H_), Internal (I_L_ vs. I_H_), and Electrode (F3/z/4, FC3/z/4, C3/z/4, CP3/z/4, P3/z/4 vs. PO3/z/4) factors on each of the SP3, SP3/P2, P2 and P3 components. The significance level was set at 0.050 for the full model. Bonferroni adjustments were applied to individual pair-wise comparison for the significant interaction effect. Pearson correlation was used to test the relationships between the amplitude change and the NRS rating change for each component. Amplitude (or NRS) change was computed by subtracting the mean amplitude (or NRS) of the control condition from those of the experimental external and internal conditions.

## Results

Two participants failed to achieve an average of 80% accuracy identifying both the nociceptive and subnociceptive stimuli in the training. They were excluded from the data analysis. Another participant was also excluded as the EEG data did not have the quality for meaningful interpretation. The final sample size for entering into the analysis was 19 participants.

### NRS Ratings

The External and Internal effects on participants’ NRS ratings were statistically significant (*P* ≤ 0.001 and 0.012, respectively); however, their interaction effects were not significant (*P* = 0.789; Table 2). For the External factor, the NRS ratings were significantly higher for the more salient stimuli (Mean = 5.1) than that for the less salient stimuli (Mean = 2.9; Table 3). For the Internal factor, on the contrary, the NRS ratings for the less salient stimuli (Mean = 4.1) were significantly higher than those for the more salient stimuli (Mean = 3.9; Table 3).

**Table 2:** Tests of within-subject effects for NRS scores and mean amplitudes of event-related potential (ERP) components.

		df	*F*	*p*-values
NRS Scores	External	1	211.479	<0.001
	Internal	1	7.775	0.012
	External × Internal	1	0.074	0.789
SP3	Electrode	2	11.317	<0.001
	Internal	2	0.291	0.749
	External	1	24.641	<0.001
	Electrode × Internal	28	3.032	<0.001
	Electrode × External	3	9.610	<0.001
	External × Internal	1	5.797	0.016
	Electrode × External × Internal	28	0.902	0.612
SP3/P2	Electrode	2	23.151	<0.001
	Internal	2	1.346	0.273
	External	1	5.335	0.033
	Electrode × Internal	28	1.621	0.024
	Electrode × External	3	5.091	0.006
	External × Internal	1	4.947	0.025
	Electrode × External × Internal	28	0.967	0.516
P2	Electrode	3	21.411	<0.001
	Internal	2	0.368	0.695
	External	1	1.770	0.200
	Electrode × Internal	28	1.178	0.244
	Electrode × External	3	4.586	0.009
	External × Internal	1	3.695	0.059
	Electrode × External × Internal	28	2.208	<0.001
P3	Electrodes	3	17.259	<0.001
	Internal	2	0.859	0.432
	External	1	20.963	<0.001
	Electrodes * Internal	28	1.588	0.030
	Electrodes * External	3	3.870	0.023
	Internal * External	1	1.550	0.231
	Electrodes * Internal * External	28	2.374	<0.001

*Note: NRS, Numeric Rating Scale which yields scores reflect the pain intensity felt by the participants from nociceptive external stimulus at the beginning of the trial (S1)*.

**Table 3:** Mean (SD) of behavioral results.

		External		
		E_L_	E_H_	Mean
**Internal**	**I_L_**	3.0 (0.2)	5.3 (0.2)	4.1 (0.2)
	**I_H_**	2.7 (0.2)	5.0 (0.2)	3.9 (0.2)
	**Mean**	2.9 (0.2)	5.1 (0.2)	

*Note: E_L_ refers to low salience external stimuli; E_H_ refers to high salience external stimuli. I_L_ refers to low salience internal sub-nociceptive images; I_H_ refers to high salience internal sub-nociceptive images. NRS, Numeric Rating Scale which yields scores reflect the pain intensity felt by the participants from the external stimuli*.

### ERP Results

#### FCN

##### SP3 (128–152 ms)

**Figure 3:**
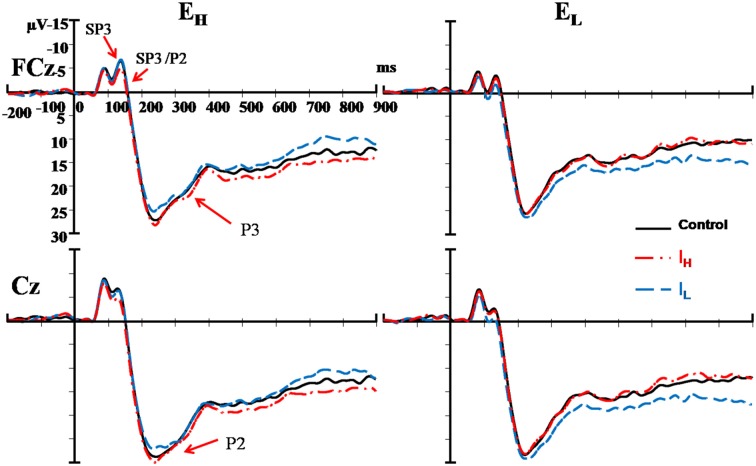
Comparisons of ERPs among the two external nociceptive stimulus and two internal sub-nociceptive image conditions recorded at FCz and Cz. Left panel presents results of high salience external condition (E_H_); and Right panel presents results of low salience external condition (E_L_). Red line: high salience internal condition (I_H_); Blue line: low salience internal condition (I_L_); and Black line: control condition.

**Figure 4:**
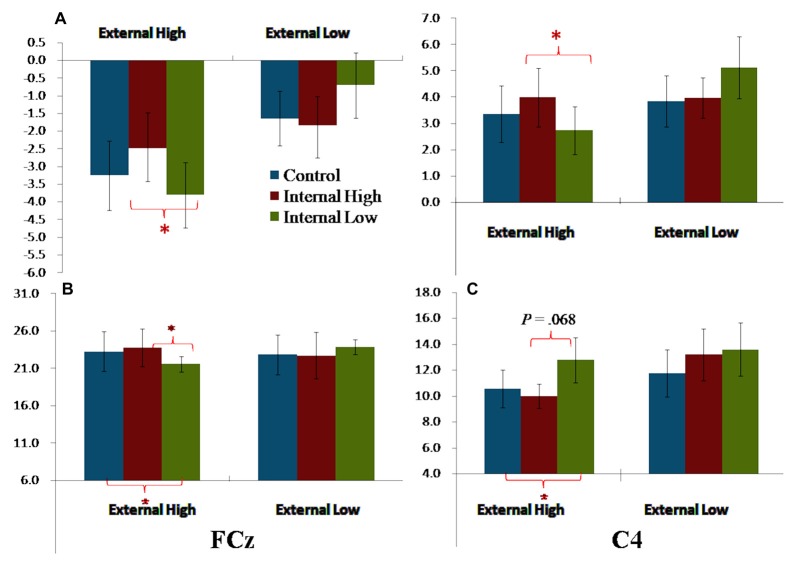
Bar charts summarizing the external-to-internal interactive effects for FCN, P2 and P3. **(A)** Left—SP3 based on average amplitudes; Right—SP3/P2 based on average amplitudes. **(B)** P2 elicited FCz. **(C)** P3 elicited at C4. Note: error bars are standard errors. Asterisks refer to *P* < 0.050.

The External and Internal interaction effect was found significant (*P* < 0.050; Table 2). In the highly salient External (E_H_) condition, mean amplitude of SP3 was significantly more negative-going in the disengagement in a low salience Internal (I_L_) condition (Mean = −3.81 μV) than that in a highly salient Internal (I_H_) condition (Mean = −2.49 μV; *P* < 0.050; Figure 3). Amplitude differences between the I_H_ and I_L_ conditions were not significant in the E_L_ condition. Amplitudes of SP3 were the most negative-going at FC4 (Mean = −5.10 μV; *P* < 0.050). The External effect was also found significant (*P* ≤ 0.001), of which the amplitudes elicited by the E_H_ condition (Mean = −3.18 μV) were significantly higher than those elicited by the E_L_ condition (Mean = −1.39 μV; *P* < 0.001; Figure 4). Other main and interaction effects were not significant.

##### SP3/P2 (152–180 ms)

The results pattern from ANOVA for the SP3/P2 time window was similar to that for the SP3 time window (Figure 4). The External and Internal interaction effects were found significant (*P* < 0.050; Table 2). In the highly salient External (E_H_) condition, mean amplitude of SP3 was significantly more negative-going in the disengagement in the low salience Internal (I_L_) condition (Mean = 2.74 μV) than that in highly salient Internal (I_H_) condition (Mean = 3.99 μV; *P* < 0.050). In the low salience External (E_L_) condition, mean amplitude of SP3 was significantly less negative-going in the disengagement in the low salience Internal (I_L_) condition (Mean = 5.13 μV) than when disengaging to the highly salient Internal (I_H_) condition (Mean = 3.86 μV; *P* < 0.050; Figure 3). Amplitude of SP3/P2 was the most negative-going at F4 (Mean = −0.60 μV) and most positive-going in Cz (Mean = 9.61 μV; *P*s < 0.050). The External effect was also found significant (*P* < 0.001), of which the amplitudes elicited by the E_H_ condition (Mean = 3.37 μV) were significantly less negative-going than those elicited by the E_L_ condition (Mean = 4.32 μV; *P* < 0.001). Other main and interaction effects were not significant.

#### P2 (200–260 ms)

The Electrode, External and Internal interaction effects were found significant (*P* < 0.001; Table 2; Figure 4). In the highly salient External (E_H_) condition, mean amplitude of P2 was significantly less positive-going in the shifting in the low salience Internal (I_L_) condition (at FCz: Mean = 21.58 μV) than when shifting to the highly salient Internal (I_H_) condition (at FCz: Mean = 23.79 μV; *P* < 0.050) or to the control condition (at FCz: Mean = 23.26 μV; *P* < 0.050). Similar patterns of results were found in electrodes F3, Fz, F4, FC4, Cz and CPz (*P*s < 0.050; Figure 3). Amplitude differences between the I_H_ and I_L_ conditions were not significant in the E_L_ condition. Amplitudes of P2 were the most positive-going at the central site (Cz: Mean = 25.61 μV; *P*s < 0.050). Other main and interaction effects were not significant.

#### P3 (320–380 ms)

The Electrode, External and Internal interaction effects were found significant (*P* < 0.001; Table 2; Figure 4). In the highly salient External (E_H_) condition, mean amplitude of P3 was marginally more positive-going in the reengagement in the low salience Internal (I_L_) condition (at C4: Mean = 12.79 μV) than in the highly salient Internal (I_H_) condition (at C4: Mean = 10.00 μV; *P* = 0.067) or significantly more positive-going in the control condition (at C4: Mean = 10.58 μV; *P* = 0.050; Figure 3). No significant interaction of External and Internal conditions could be found in other electrodes, and amplitude differences between the I_H_ and I_L_ conditions were not significant in the E_L_ condition. Amplitudes of P3 were the most positive-going at the fronto-central site (FCz: Mean = 15.52 μV; *P*s < 0.050). The Electrode and Internal interaction effects were found significant (*P* < 0.050). At C4, mean amplitude of P3 was significantly more positive-going in reengagement in the low salience Internal (I_L_) condition (Mean = 13.20 μV) than in the highly salient Internal (I_H_) condition (Mean = 11.62 μV; *P* < 0.050) or in the control condition (Mean = 11.18 μV; *P* < 0.001). The Electrode and External interaction effects were found significant (*P* < 0.050), of which the amplitudes elicited by the E_H_ condition were significantly more positive-going than those elicited by the E_L_ condition at all 14 electrodes except at F3 (*P*s < 0.050). The External effect was also found significant (*P* < 0.001), of which the amplitudes elicited by the E_H_ condition (Mean = 12.19 μV) were significantly more positive-going than those elicited by the E_L_ condition (Mean = 10.39 μV; *P* < 0.001). Other main and interaction effects were not significant.

### Correlations between Changes in NRS Ratings and ERP Amplitudes

Among the four external-to-internal conditions, significant correlations were only revealed in the E_H_/I_L_ condition between changes in the amplitudes and those in the NRS ratings. Changes in the amplitudes of the P3 (I_L_ minus control) recorded at the centro-parietal electrodes (C3, C4, CP3, CP4 and CPz) were positively and moderately correlated with changes (I_L_ minus control) in the NRS scores (*r* = 0.537 [at CP3] to 0.638 [at CPz], *P*s < 0.050).

## Discussion

The present study investigated the characteristics of the processes associated with orienting attention from external nociceptive stimuli to internal subnociceptive images. The elicitation of FCN, P2 and P3 supported the hypothesized disengaging, shifting and reengagement subprocesses. The significant findings in the amplitude change of these components observed in the high but not in the low salience external stimulus conditions suggest interactions of both bottom-up and top-down processes in the external-to-internal orienting attention. The bottom-up process would be primarily stimuli driven, represented by the FCN, whereas the top-down process would be primarily goal directed, represented by the P2 and P3 components.

### Disengagement from External Stimuli

The fronto-centrally distributed FCN elicited by the nociceptive stimuli is consistent with that reported in Dowman (2007, 2011). This component has been associated with a stimulus-driven, bottom-up process in which a higher level of attention was found allocated to higher rather than lower salient nociceptive stimuli. Besides, the FCN was associated with visualization of somatosensory stimuli in working memory (Legrain et al.’s 2013) suggesting that the disengagement from the nociceptive stimuli would have involved working memory, particularly those of high salience. Different from previous studies, we manipulated both the external and internal conditions by classifying stimuli/images in both environments into high and low salience levels. The significant Condition × Salience on the FCN amplitudes indicated the disengagement subprocess was modulated by the highly salient nociceptive stimuli. Highly salient external stimuli resulted in larger incongruence in intensities between the external stimuli and internal image representations than the low salience stimuli. The larger incongruence would have generated larger mental conflicts and hence stronger top-down cognitive control for resolving them (Kerns et al., 2004; Egner et al., 2005).

### External-to-Internal Shifting

Similar to FCN, this study revealed a significant external-to-internal interaction effect on the fronto-centrally distributed P2. The P2 has been previously associated with the shifting attention process involving somatosensory stimuli (Legrain et al.’s 2013). Nevertheless, the less positive-going P2 yielded in this study is inconsistent with that reported in Chan et al.’s (2012). Chan et al. yielded more positive-going P2 when participants shifted their attention from nociceptive stimuli to self-generated subnociceptive images. An analysis of the task designs among these three studies suggests that the discrepancy in the results is likely to be attributable to whether the somatosensory images to be generated are anticipatory or contingent to the perceived nociceptive stimuli. In Chan et al.’s (2012) study, the participants were to generate subnociceptive images at the salient levels contingent upon those of the nociceptive stimuli perceived at the beginning of the trial. Participants in Legrain et al.’s (2013) study were required to generate dot images, as to generate subnociceptive images in our study, which had been maintained in the working memory throughout the trial. It is therefore plausible that when compared with Chan et al.’s (2012) study, our participants would have involved more top-down influence for generating the subnociceptive images, which evoked less positive-going P2 amplitudes.

It is noteworthy that the modulation effect on P2 was only observed in the high but not in the low salience external condition stimuli and when the external-to-internal incongruence was large. It is plausible that the significant P2 reflected a top-down process that interacted with the prior bottom-up, stimulus-driven process (perception of nociceptive stimulation). In fact, interactions between the top-down and bottom-up processes modulating shifting attention have been reported in behavioral studies on visual perception (Caparos and Linnell, 2010; Linnell and Caparos, 2011). The results of our study provide evidence that such interactions occurred at around 200–260 ms after presentation of the external somatosensory stimulus represented by the fronto-central P2.

### Reengagement

Centrally distributed P3a was suggested to reflect reengaging attention (Dowman, 2011). The significant P3 results obtained in this study can be interpreted as participants reengaged with the internal subnociceptive images after shifting from the external nociceptive stimuli. The marginal significance (*P* = 0.067) yielded for the P3 effects suggests caution should be taken when interpreting such effects. Significant correlations were revealed between the changes in the P3 amplitudes at the central and parietal electrodes (C3, C4, CP3, CP4 and CPz) and the attenuation on the perceived pain intensity felt for the external nociceptive stimulus. The attenuation was based on scores assigned by the participants using an NRS of the recall of the nociceptive sensation after generating the internal sub-nociceptive by end of the trial. In other words, less positive-going P3 amplitudes were correlated with larger attenuation of the pain intensity scores. The attenuation effect was only observed in trials involving high salience external stimuli and generating a low salience internal image. Previous studies reported that anterior P3 was related to reengaged attention with external nociceptive stimulus (Dowman, 2007, 2011; Dowman et al., 2007), and the posterior P3 was associated with rehearsal of the mental representation (Donchin, 1981; Pontifex et al., 2009). Our findings offer a plausible mechanism for explaining the attenuation effects on pain intensity after orienting attention, such as those reported in Fors et al. (2002) and Chan et al.’s (2012), and that such effects would be the result of a top-down-dominated reengaging process in orienting attention. The processes probably would involve mental conflict of cognitive control (Kerns et al., 2004; Egner et al., 2005).

### Limitations

This study has several limitations. First, the task design did not control the time at which the participants generated the internal subnociceptive images. This could have confounded the latency of the P3 components that impacted peak amplitude and were subsequently attributable to the marginally significant interaction between the external and internal effects. Future task design should improve the timing of generating the internal somatosensory images and perhaps increase the sample size for increasing the effect size and power of the data analysis, respectively. Second, this study was based on a somatosensory stimulus and images—the results may not be readily generalized to other sensory modalities. Further studies should be conducted to test the robustness of the external-to-internal orienting attention on visual and auditory modalities.

## Conclusion

Salience of external stimulus and internal representation was found to modulate the external-to-internal process. Perception of a high salience external stimulus exerted effects on the disengagement and shifting processes, and subsequently the reengaging process with which internal images were generated. This reengaging process was revealed to relate to the perception of the external stimulus and in this study was attenuation of the pain intensity perceived for the nociceptive sensation felt. Our findings offer a plausible mechanism for explaining attentional dysfunction among patients with chronic pain and its therapeutic intervention. Future studies should be conducted to replicate the experiment on patients with chronic pain to test this proposition.

## Author Contributions

BKHC contributed to interpretation of data and article writing. CCHC contributed to conceptualization, study design, interpretation of data, and article writing. JP contributed to conceptualization, study design, data collection, data analysis, interpretation of data, and article writing. SCCC contributed to conceptualization, study design, interpretation of data, and article writing. QY contributed to data collection, data analysis, interpretation of data, and article writing. All approved the version to be published and agreed to be accountable for all aspects of the work in ensuring that questions related to accuracy or integrity of any part of the work were appropriately investigated and resolved.

## Conflict of Interest Statement

The authors declare that the research was conducted in the absence of any commercial or financial relationships that could be construed as a potential conflict of interest.

## References

[B1] Caparos S. Linnell K. J. (2010). The spatial focus of attention is controlled at perceptual and cognitive levels. J. Exp. Psychol. Hum. Percept. Perform. 36, 1080–1107. 10.1037/a0020367 20873935

[B2] Chan S. C. C. Chan C. C. H. Kwan A. S. K. Ting K. H. Chui T. Y. (2012). Orienting attention modulates pain perception: an ERP study. PLoS One 7:e40215. 10.1371/journal.pone.0040215 22768257 PMC3387012

[B3] Chun M. M. Golomb J. D. Turk-Browne N. B. (2011). A taxonomy of external and internal attention. Ann. Rev. Psychol. 62, 73–101. 10.1146/annurev.psych.093008.100427 19575619

[B4] De Pascalis V. Cacace I. Massicolle F. (2008). Focused analgesia in waking and hypnosis: effects on pain, memory, and somatosensory event-related potentials. Pain 134, 197–208. 10.1016/j.pain.2007.09.005 18023535

[B5] Donchin E. (1981). Surprise!… Surprise? Psychophysiology 18, 493–513. 10.1111/j.1469-8986.1981.tb01815.x 7280146

[B6] Dowman R. (2007). Neural mechanisms of detecting and orienting attention toward unattended threatening somatosensory targets. I. Intermodal effects. Psychophysiology 44, 407–419. 10.1111/j.1469-8986.2007.00508.x 17371498

[B7] Dowman R. (2011). The role of somatic threat feature detectors in the attentional bias toward pain: effects of spatial attention. Psychophysiology 48, 397–409. 10.1111/j.1469-8986.2010.01068.x 20636292

[B8] Dowman R. Darcey T. Barkan H. Thadani V. Roberts D. (2007). Human intracranially-recorded cortical responses evoked by painful electrical stimulation of the sural nerve. Neuroimage 34, 743–763. 10.1016/j.neuroimage.2006.09.021 17097306

[B9] Egner T. Jamieson G. Gruzelier J. (2005). Hypnosis decouples cognitive control from conflict monitoring processes of the frontal lobe. Neuroimage 27, 969–978. 10.1016/j.neuroimage.2005.05.002 15964211

[B10] Fors E. A. Sexton H. Götestam K. G. (2002). The effect of guided imagery and amitriptyline on daily fibromyalgia pain: a prospective, randomized, controlled trial. J. Psychiatr. Res. 36, 179–187. 10.1016/s0022-3956(02)00003-1 11886696

[B11] Griffin I. C. Nobre A. C. (2003). Orienting attention to locations in internal representations. J. Cogn. Neurosci. 15, 1176–1194. 10.1162/089892903322598139 14709235

[B12] Jensen M. P. Karoly P. Braver S. (1986). The measurement of clinical pain intensity—a comparison of 6 methods. Pain 27, 117–126. 10.1016/0304-3959(86)90228-9 3785962

[B13] Katus T. Andersen S. K. Müller M. M. (2012). Maintenance of tactile short-term memory for locations is mediated by spatial attention. Biol. Psychol. 89, 39–46. 10.1016/j.biopsycho.2011.09.001 21925566

[B14] Kerns J. G. Carter C. S. MacDonald A. W. III Cho R. Y. Stenger V. A. Carter C. S. (2004). Anterior cingulate conflict monitoring and adjustments in control. Science 303, 1023–1026. 10.1126/science.1089910 14963333

[B15] Kida T. Nishihira Y. Wasaka T. Nakata H. Sakamoto M. (2004). Differential modulation of temporal and frontal components of the somatosensory N140 and the effect of interstimulus interval in a selective attention task. Cogn. Brain Res. 19, 33–39. 10.1016/j.cogbrainres.2003.10.016 14972356

[B16] Legrain V. Crombez G. Plaghki L. Mouraux A. (2013). Shielding cognition from nociception with working memory. Cortex 49, 1922–1934. 10.1016/j.cortex.2012.08.014 23026759

[B17] Lepsien J. Nobre A. C. (2006). Cognitive control of attention in the human brain: insights from orienting attention to mental representations. Brain Res. 1105, 20–31. 10.1016/j.brainres.2006.03.033 16729979

[B18] Linnell K. J. Caparos S. (2011). Perceptual and cognitive load interact to control the spatial focus of attention. J. Exp. Psychol. Hum. Percept. Perform. 37, 1643–1648. 10.1037/a0024669 21767051

[B19] Ohara S. Lenz F. A. Zhou Y. D. (2006). Modulation of somatosensory event-related potential components in a tactile-visual cross-modal task. Neuroscience 138, 1387–1395. 10.1016/j.neuroscience.2005.12.005 16442738

[B20] Petersen S. E. Posner M. I. (2012). The attention system of the human brain: 20 years after. Annu. Rev. Neurosci. 35, 73–89. 10.1146/annurev-neuro-062111-150525 22524787 PMC3413263

[B21] Pontifex M. B. Hillman C. H. Polich J. (2009). Age, physical fitness, and attention: P3a and P3b. Psychophysiology 46, 379–387. 10.1111/j.1469-8986.2008.00782.x 19170947 PMC2763440

[B22] Posner M. I. Walker J. A. Friedrich F. J. Rafal R. D. (1984). Effects of parietal injury on covert orienting of attention. J. Neurosci. 4, 1863–1874. 6737043 10.1523/JNEUROSCI.04-07-01863.1984 PMC6564871

[B23] Sauce B. Wass C. Smith A. Kwan S. Matzel L. D. (2014). The external-internal loop of interference: two types of attention and their influence on the learning abilities of mice. Neurobiol. Learn. Mem. 116, 181–192. 10.1016/j.nlm.2014.10.005 25452087 PMC5000557

[B24] Staines W. R. Popovich C. Legon J. K. Adams M. S. (2014). Early modality-specific somatosensory cortical regions are modulated by attended visual stimuli: interaction of vision, touch and behavioral intent. Front. Psychol. 5:351. 10.3389/fpsyg.2014.00351 24795684 PMC4006034

[B25] Tanoue R. T. Jones K. T. Peterson D. J. Berryhill M. E. (2012). Differential frontal involvement in shifts of internal and perceptual attention. Brain Stimul. 6, 675–682. 10.1016/j.brs.2012.11.003 23266133 PMC3608701

[B26] Williamson A. Hoggart B. (2005). Pain: a review of three commonly used pain rating scales. J. Clin. Nurs. 14, 798–804. 10.1111/j.1365-2702.2005.01121.x 16000093

